# Influence of Chemokine N-Terminal Modification on Biased Agonism at the Chemokine Receptor CCR1

**DOI:** 10.3390/ijms20102417

**Published:** 2019-05-15

**Authors:** Julie Sanchez, J. Robert Lane, Meritxell Canals, Martin J. Stone

**Affiliations:** 1 Infection and Immunity Program, Monash Biomedicine Discovery Institute, and Department of Biochemistry and Molecular Biology, Monash University, Clayton, VIC 3800, Australia; julie.sanchez@nottingham.ac.uk; 2 Centre for Membrane Proteins and Receptors, Nottingham University, Nottingham, NG7 2RD, UK; rob.lane@nottingham.ac.uk

**Keywords:** chemokine, chemokine receptor, chemokine receptor 1 (CCR1), G protein-coupled receptor (GPCR), binding, receptor activation, biased agonism

## Abstract

Leukocyte migration, a hallmark of the inflammatory response, is stimulated by the interactions between chemokines, which are expressed in injured or infected tissues, and chemokine receptors, which are G protein-coupled receptors (GPCRs) expressed in the leukocyte plasma membrane. One mechanism for the regulation of chemokine receptor signaling is biased agonism, the ability of different chemokine ligands to preferentially activate different intracellular signaling pathways via the same receptor. To identify features of chemokines that give rise to biased agonism, we studied the activation of the receptor CCR1 by the chemokines CCL7, CCL8, and CCL15(Δ26). We found that, compared to CCL15(Δ26), CCL7 and CCL8 exhibited biased agonism towards cAMP inhibition and away from β-Arrestin 2 recruitment. Moreover, N-terminal substitution of the CCL15(Δ26) N-terminus with that of CCL7 resulted in a chimera with similar biased agonism to CCL7. Similarly, N-terminal truncation of CCL15(Δ26) also resulted in signaling bias between cAMP inhibition and β-Arrestin 2 recruitment signals. These results show that the interactions of the chemokine N-terminal region with the receptor transmembrane region play a key role in selecting receptor conformations coupled to specific signaling pathways.

## 1. Introduction

A defining feature of inflammatory responses is the trafficking of leukocytes into the affected tissues. Leukocyte trafficking is stimulated and regulated by the interactions of chemokines—small proteins expressed at the site of tissue injury—with chemokine receptors, G protein-coupled receptors (GPCRs) expressed in the leukocyte plasma membrane [1,2,3]. The genomes of humans and other mammals each encode approximately 50 chemokines and approximately 20 chemokine receptors. Different classes of leukocytes express distinct arrays of chemokine receptors, and chemokines are differentially expressed in tissues as a response to inflammatory stimuli. Moreover, most chemokines activate multiple receptors and most receptors respond to numerous chemokines. These factors result in immensely complex potential networks of chemokine-stimulated receptor activation and leukocyte recruitment.

Chemokines are classified into two major subfamilies (CCL and CXCL; L indicates ligand) and two minor subfamilies (XCL and CX_3_CL) according to the spacing between the first two of four conserved cysteine residues. The chemokine receptors are similarly classified (CCR, CXCR, XCR, and CX_3_CR; R indicates receptor) according to the subfamily of chemokines for which they are selective. Here we focus on CCR1, a CC chemokine receptor expressed on peripheral blood neutrophils, monocytes, and macrophages [4] as well as natural killer cells and immature myeloid cells [5,6]. CCR1 is activated by numerous CC chemokines [7] and has been implicated in the pathology of various inflammatory diseases [8,9,10,11,12,13,14,15], although clinical trials targeting CCR1 have not yet yielded successful anti-inflammatory drugs [16], a problem also encountered for trials targeting many other chemokine receptors [17].

The lack of success in trials of anti-inflammatory drugs targeting chemokine receptors can be attributed in part to the complex regulation of chemokine–receptor networks. These networks can be regulated on numerous levels, including gene expression, alternative splicing, partial proteolysis, various other post-translational modifications, control of stability or localization, and competition with active or decoy receptors [18,19]. Moreover, it is now well established that, like other GPCRs, chemokine receptors are able to stimulate different intracellular signaling pathways (and therefore cellular outcomes) when activated by different chemokine ligands, a phenomenon known as biased agonism.

Biased agonism has been observed for several chemokine receptors, including CXCR2, CXCR3, CCR1, CCR2, CCR4, CCR5, CCR7, and CCR10 [20,21,22,23]. In particular, Rajagopal et al. compared the abilities of several chemokine ligands to activate CCR1, giving rise to inhibition of cAMP signaling, recruitment of β-Arrestin (βArr), and internalization of the receptor [20]. They observed, for example, that, when compared to the chemokine CCL3 as a reference, the chemokines CCL5 and CCL23 displayed preferential activation of the βArr pathway relative to the cAMP (Gα_i_-coupled) pathway.

**Figure 1:**
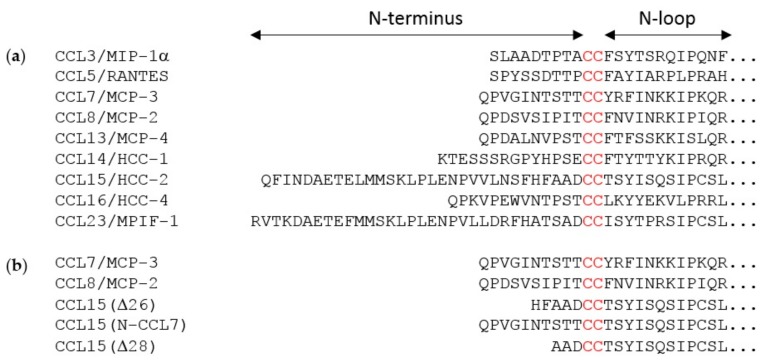
(**a**) Partial sequences of nine cognate chemokine ligands for the receptor CCR1; and (**b**) partial sequences of the chemokines used in this study. The conserved CC motif is in red. The arrows at the top indicate the N-terminal and N-loop regions, which participate in receptor recognition.

The ability of different cognate chemokines to induce distinct intracellular signals via CCR1 is expected to be related to the amino acid sequence variation in regions of these chemokines known to interact with the receptor (Figure 1a). The N-loop regions of chemokines interact with the extracellular N-terminal regions of their receptors, and are thought to contribute primarily to binding affinity, whereas the N-terminal regions of chemokines insert amongst the transmembrane (TM) helices of the receptors, thus affecting both binding affinity and TM signaling [24,25,26,27,28]. Therefore, considering the substantial sequence variation in the N-terminal regions of CCR1-cognate chemokines (Figure 1a), we postulated that the N-terminus would influence biased agonism at CCR1.

Here, we have systematically compared the CCR1-mediated signaling profiles of the CCR1-cognate chemokines CCL7 (previously called monocyte chemoattractant protein-3, MCP-3), CCL8 (MCP-2), and CCL15 (hemofiltrate CC chemokine-2, HCC-2). We found that CCL7 and CCL8 displayed significant signaling bias towards cAMP pathway and away from βArr2 pathway in comparison to CCL15. To elucidate the role of the chemokine N-terminus on the observed bias, we further examined CCL15 mutants with variations of N-terminal sequences and lengths. We found that both of these factors have effects on signaling bias. Our results have implications for mechanistic models of chemokine receptor activation. 

## 2. Results

### 2.1. Receptor Binding and Activation of CCR1 by Wild Type Chemokines

**Figure 2:**
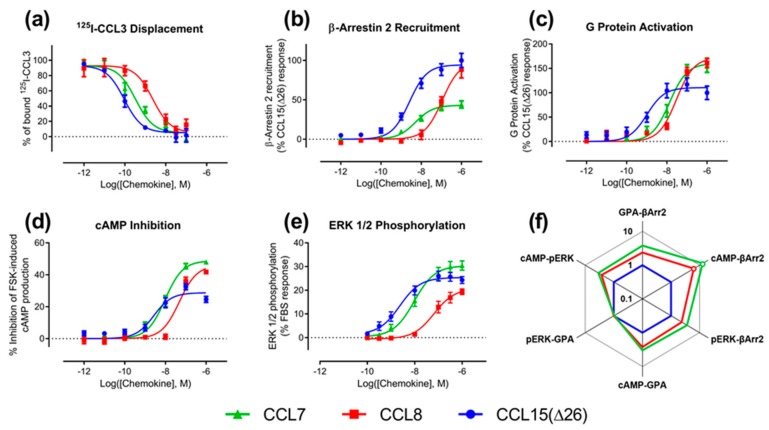
CCR1 binding and activation by wild type chemokines. Each panel (**a**–**e**) shows the concentration-response data for a different binding or signaling readout, measured as described in Materials and Methods: (**a**) binding, (**b**) βArr2 recruitment, (**c**) G protein activation, (**d**) inhibition of forskolin-induced cAMP production, and (**e**) ERK1/2 phosphorylation. In panels (**a**–**e**), data points represent means ± SEM of at least three independent experiments performed in duplicate. (**f**) Web of bias for wild type chemokines at CCR1. Bias factors were calculated as described in Materials and Methods. Each axis represents the 10^ΔΔ(Log(τ/K^_A_^))^ values comparing the two indicated pathways. Open circles indicate significant differences between values of ΔLog(τ/K_A_) determined at different pathways for a particular ligand, determined by two-way ANOVA with a Tukey’s multiple-comparison test (*p* < 0.05).

The flexible N-terminal regions, preceding the conserved CC or CXC motif, of most chemokines consist of ~8–10 amino acid residues. This enables the chemokine N-terminal region, in a largely extended conformation, to occupy the majority of space within the binding pocket defined by the TM helices of chemokine receptors. However, two of the cognate ligands of CCR1 (CCL15 and CCL23) have much longer N-terminal regions (31 and 32 residues, respectively). Full-length CCL15 is reported to have low potency of CCR1 activation, but a form of CCL15 with the first 26 residues removed, CCL15(Δ26), has much higher affinity and potency at CCR1, and further truncation results in moderate decreases in affinity and potency relative to CCL15(Δ26) [29]. These shorter forms of CCL15 contain only five or fewer residues in their N-terminal regions, insufficient to occupy the TM binding pocket, raising the question of whether they achieve receptor activation by a different structural mechanism from other chemokines, potentially giving rise to biased agonism. To investigate this, we compared CCR1 binding and activation by three human chemokines: CCL7, CCL8, and CCL15(Δ26) (Figure 1b and Figure 2). 

We determined the binding affinities of chemokines for CCR1 expressed in Flp-In T-REx human embryonic kidney (HEK) 293 cells, using a radioligand displacement assay (Figure 2a and Table 1). All three chemokines competed with ^125^I-labeled CCL3 for binding to CCR1 in a concentration-dependent manner. The data indicated that CCL15(Δ26) had the highest affinity, with half-maximal displacement at a concentration (*IC_50_*) of 0.093 nM, whereas CCL7 and CCL8 had lower affinities with *IC_50_* values of 0.33 and 2.3 nM, respectively (Table 1).

**Figure A1:**
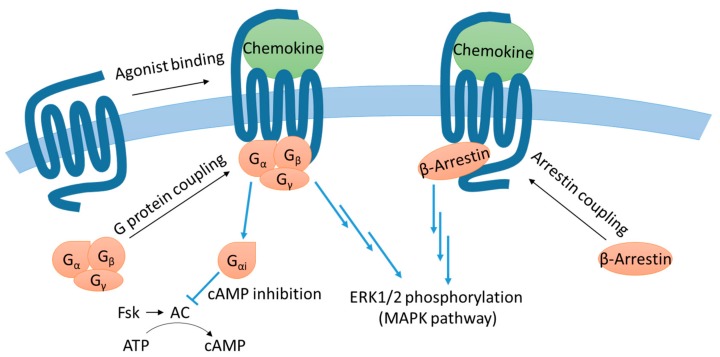
Simplified diagram of the signaling assays measured. Chemokine recognition induces conformational change(s) in the receptor. The ensemble of activated GPCR conformations is dependent on the agonist bound on the extracellular side and determines which intracellular effectors, such as G proteins or Arrestins, are preferentially recognized to induce further downstream signaling. Common GPCR signaling pathways include ERK1/2 phosphorylation through activation of the mitogen activated protein kinases (MAPK) pathway and Gα_i_-mediated cAMP inhibition following trimeric G protein dissociation. The specific pathway giving rise to ERK1/2 phosphorylation may vary depending on the cellular context. Here we used forskolin (Fsk) to directly stimulate cAMP production via activation of adenylyl cyclase (AC).

**Figure A2:**
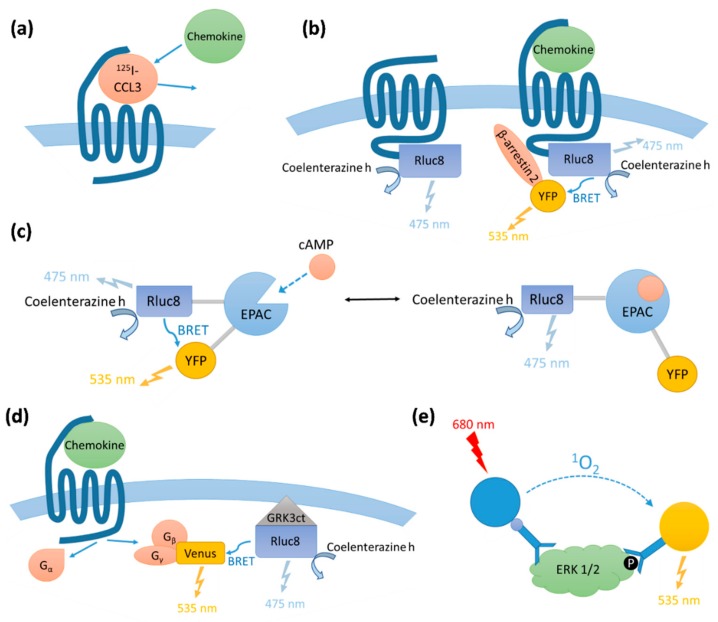
Experimental design for binding and signaling assays. Each panel shows a schematic representation of the assays described in the Materials and Methods: (**a**) competitive radioligand binding measurement using ^125^I-CCL3 as a probe, (**b**) β-Arrestin2-YFP recruitment to CCR1-Rluc8, (**c**) inhibition of forskolin-induced cAMP production, with cAMP monitored using the guanine nucleotide exchange protein activated by cAMP (EPAC) linked to a BRET donor and acceptor, (**d**) G protein activation via dissociation of the βγ-Venus subunit, and (**e**) AlphaScreen-based ERK phosphorylation measurement.

We compared the abilities of CCL7, CCL8, and CCL15(Δ26) to activate CCR1 using four different cell-based signaling assays, as measured 5–10 min after agonist stimulation (Figure A1 and Figure A2). Recruitment of βArr is a proximal (non-amplified) measure of receptor activation, whereas G_i1_ protein activation, and the downstream signals of inhibition of cAMP production and phosphorylation of extracellular signal-regulated kinases 1 and 2 (ERK1/2), are all amplified to varying degrees. In all assays, all three chemokines stimulated concentration-dependent signaling via CCR1 (Figure 2b–e and Table 1). In the recruitment of βArr, CCL15(Δ26) and CCL7 had similar potencies (*pEC_50_*), but CCL15(Δ26) exhibited a significantly higher maximal effect (*E_max_*) than CCL7 (Figure 2b and Table 1). In contrast, CCL8 had lower potency than the other two chemokines but its maximal effect was similar to that of CCL15(Δ26). In an initial indication of biased agonism, we observed that the order of potencies and maximal effects was not the same in all assays (Table 2). For example, in all assays, CCL15(Δ26) displayed higher potency than CCL8, but the potency of CCL7 was similar to that of CCL8 in the G protein activation assay and similar to that of CCL15(Δ26) in the other three assays. In addition, CCL15(Δ26) displayed a significantly higher maximal effect than CCL7 in the βArr recruitment assay, but significantly lower maximal effect than CCL7 in both G protein activation and cAMP inhibition assays.

To detect and quantify biased agonism at CCR1, we analyzed the data for each concentration-response experiment using a derivation of the Black and Leff operational model of agonism [30,31]. This analysis yielded a “transduction coefficient”, log(τ/K_A_), as a measure of intrinsic activity of an agonist at a given pathway, which was normalized relative to a reference ligand, CCL15(Δ26). Comparison of the normalized transduction coefficients across the different signaling pathways (Figure 2f, Table A1) revealed that CCL7 and CCL8 displayed biased agonism relative to CCL15(Δ26) with both chemokines showing bias towards cAMP inhibition and away from β-Arrestin 2 recruitment compared to CCL15(Δ26). 

**Figure A3:**
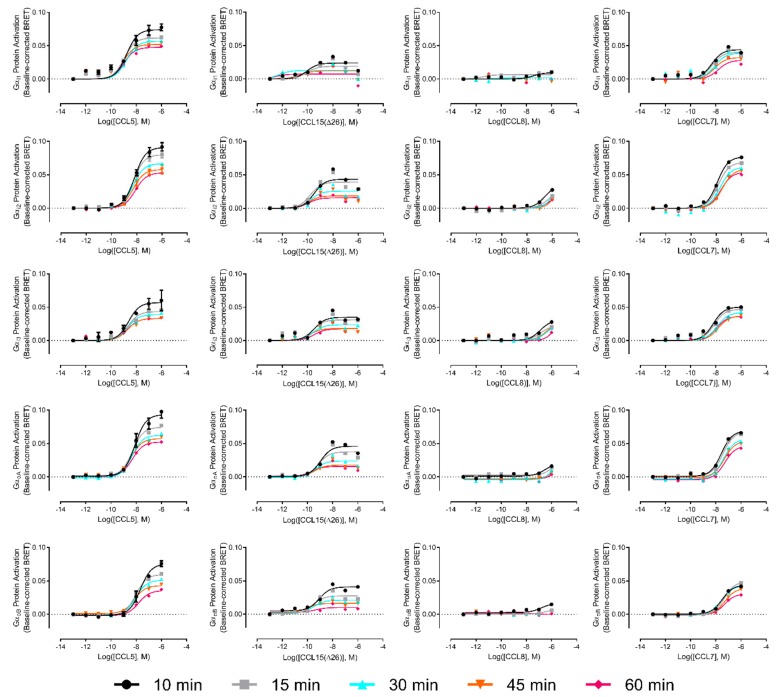
G protein activation concentration-response curves and time courses. Each graph shows the concentration-response data for a different chemokine and a different Gα protein. Each column shows a different chemokine: (left to right) CCL5, CCL15(Δ26), CCL7, and CCL8 from. Each row shows a different Gα protein: (top to bottom) i1, i2, i3, oA, and oB from. Data points represent means ± SEM of at least three independent experiments performed in duplicate.

In addition to identifying significant biased activation of cAMP inhibition versus βArr pathways, the above analysis showed that, in comparison to CCL15(Δ26), CCL7 and CCL8 displayed a slight preference for stimulation of the cAMP inhibition response over both the ERK1/2 phosphorylation response and the G protein activation response. Although these comparisons did not reach statistical significance, they suggested that CCR1 might preferentially associate with certain G protein subtypes in a biased manner when activated by different ligands. Therefore, to investigate the possibilities of G protein subtype-coupling bias, we repeated the G protein activation assay using a set of five Gα subunits known to inhibit adenylyl cyclase and interact with chemokine receptors (i1, i2, i3, oA, and oB). Moreover, we recognized that the activation of different G proteins subtypes could potentially follow distinct time courses, thereby resulting in measurable bias when detected at certain time points. We thus used detection times of 10, 15, 30, 45, and 60 min after agonist stimulation. The concentration-response curves are presented in the Appendix A (Figure A3). Gα_i1_ had the best coupling to CCR1, irrespective of the ligand used, although saturation was not reached for CCL8. CCL15(Δ26) was the most potent ligand and CCL5 was the most efficacious ligand, irrespective of the Gα protein subtype. The maximum response was observed 10 min after the addition of ligands and *E_max_* values decreased overtime. We did not observe any significant bias between activation of different Gα subtypes at any of the time points investigated.

### 2.2. Influence of Chemokine N-Terminal Sequence on Biased Agonism at CCR1

**Figure 3:**
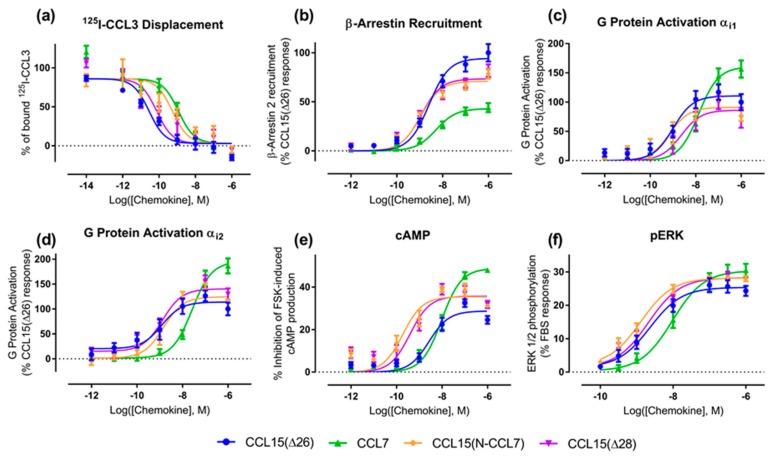
CCR1 binding and activation by chemokine N-terminal variants. Each graph shows the concentration-response data for a different signal readout, as described in Materials and Methods: (**a**) binding, (**b**) βArr2 recruitment, (**c**) G protein activation using Gα_i1_, (**d**) G protein activation using Gα_i2_, (**e**) inhibition of forskolin-induced cAMP production, and (**f**) ERK1/2 phosphorylation. Data points represent means ± SEM of at least three independent experiments performed in duplicate.

**Figure 4:**
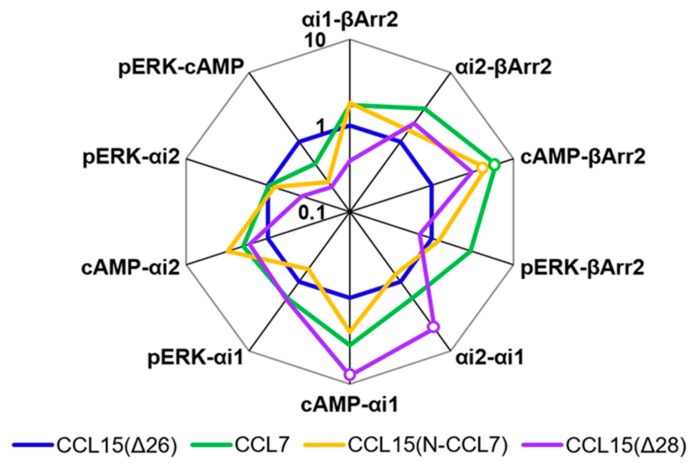
Web of bias for chemokine N-terminal variants at CCR1. Bias factors were calculated as described in Materials and Methods. Each axis represent the 10^ΔΔ(Log(τ/K^_A_^))^ values comparing two particular pathways. Data points represent the mean of at least three independent experiments performed in duplicate. Open circles indicate significant differences between values of ΔLog(τ/K_A_) for different pathways for a particular ligand, determined by two-way ANOVA with a Tukey’s multiple-comparison test (*p* < 0.05).

**Figure A4:**
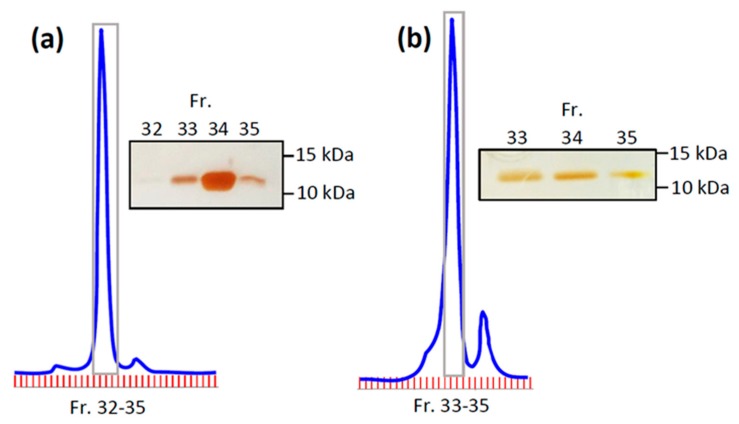
Purification of CCL15 chimeras (**a**) CCL15(N-CCL7) and (**b**) CCL15(Δ28). Each panel shows the preparative size exclusion chromatogram in dark blue and SDS-PAGE gel under non-reducing conditions of the selected fractions (indicated in light grey boxes on the chromatograms).

To test the hypothesis that the N-terminal region is the primary site influencing biased agonism at CCR1, we generated a chimeric chemokine consisting of CCL15 with the N-terminal region substituted by that of CCL7, named CCL15(N-CCL7) (Figure 1b and Figure A4a). CCL15(N-CCL7) bound to CCR1 with affinity indistinguishable from that of CCL7, which was significantly lower than the affinity of CCL15(Δ26), indicating that the N-terminal region of CCL15(Δ26) contributes to its increased CCR1 binding affinity (Figure 3a, Table 3). However, the effects of N-terminal substitution on CCR1 activation were different for the various functional assays (Figure 3b–f, Table 3). In both G protein activation (using Gα_i1_ or Gα_i2_) and ERK1/2 phosphorylation assays, the chimeric chemokine displayed concentration-response profiles similar to those of CCL15(Δ26), suggesting that the N-terminal regions of the two chemokines are equally capable of activating these pathways. In the βArr recruitment assay, CCL15(N-CCL7) displayed a maximal effect intermediate between the two parental chemokines, indicating that the N-terminal regions of the two chemokines contribute differentially to βArr recruitment. In the cAMP inhibition assay, CCL15(N-CCL7) displayed significantly higher potency than either CCL15(Δ26) or CCL7, suggesting that the N-terminus of CCL7 and other regions of CCL15(Δ26) were able to cooperatively and selectively stabilize the conformation of CCR1 giving rise to cAMP inhibition. Analysis of transduction coefficients for CCR1 activation indicated that, relative to CCL15(Δ26), CCL15(N-CCL7) is biased towards cAMP inhibition and away from βArr recruitment (Figure 4, Table A2). Although the bias profile of CCL15(N-CCL7) was similar to that of CCL7, the concentration-response curves indicate that the underlying causes of bias may be different for these two proteins. 

### 2.3. Influence of CCL15 N-Terminal Length on Biased Agonism at CCR1

A previous study showed that truncation of the CCL15(Δ26) N-terminus by two additional residues, to CCL15(Δ28), resulted in a loss of affinity and a ~3-fold reduction of potency for CCR1 activation, as measured using an aequorin luminescence assay, which senses changes in intracellular Ca^2+^ concentration [29]. To investigate whether CCL15 truncation differentially influenced activation of various signaling pathways via CCR1, we expressed and purified CCL15(Δ28) (Figure 1b and Figure A4b) and compared its CCR1 binding and activation to those of CCL15(Δ26). In membrane preparations of cells expressing CCR1, CCL15(Δ28) and CCL15(Δ26) bound to CCR1 with similar affinities (Figure 3a, Table 3). However, N-terminal truncation resulted in different effects for the various signaling assays (Figure 3b–f, Table 3). In the ERK1/2 phosphorylation assay, the concentration-response profiles of CCL15(Δ28) and CCL15(Δ26) were indistinguishable. In the G protein activation assays, the two chemokines displayed similar potency but the maximal effect of CCL15(Δ28) was slightly lower than that of CCL15(Δ26) when Gα_i1_ was used and slightly higher than that of CCL15(Δ26) when Gα_i2_ was used, albeit not reaching statistical significance. In the βArr recruitment assay, CCL15(Δ28) displayed similar potency but slightly lower maximal effect than CCL15(Δ26), whereas in the cAMP inhibition assay, CCL15(Δ28) displayed significantly higher potency relative to CCL15(Δ26). Analysis of transduction coefficients showed that, relative to CCL15(Δ26), CCL15(Δ28) is biased towards Gα_i2_ activation and away from Gα_i1_ activation and towards cAMP inhibition and away from Gα_i1_ activation (Figure 4, Table A2).

## 3. Discussion

The data presented here show that CCR1 is differentially activated by the cognate chemokine ligands CCL7, CCL8, and CCL15(Δ26). Specifically, whereas the order of ligand potency in all assays essentially reflects the relative affinities of the three ligands for CCR1, CCL15(Δ26) stimulates βArr recruitment with higher maximal effect than CCL7 and gives rise to inhibition of cAMP synthesis with lower maximal effect than both these other chemokines. The simplest interpretation of these observations is that different CCR1 ligands can preferentially stabilize the activated conformations of CCR1 that are coupled either to βArr or to G proteins containing the Gα_i_ subunit. This conclusion is also consistent with a previous report showing that treatment of CCR1 with CCL5 and CCL23 gave rise to preferential activation of the βArr pathway whereas, in comparison, CCL3 caused preferential activation of the cAMP inhibition pathway [20].

Whereas previous studies have revealed biased agonism at chemokine receptors, none of these studies has identified the structural elements of the chemokines that selectively stabilize different receptor conformations. Nevertheless, the structures of chemokine–receptor complexes have confirmed that the flexible N-terminal regions of chemokines bind into a deep pocket formed by the TM helices of the receptor [24,25]. In the case of chemokine agonists, it is generally presumed that this binding interaction stabilizes the activated conformation of the receptor. Thus, it was reasonable to hypothesize that the different N-terminal sequences of CCR1 cognate chemokines are able to selectively stabilize different activated conformations of CCR1, resulting in biased signaling. Consistent with this hypothesis, we found that substituting the N-terminal region of CCL15(Δ26) with that of CCL7 resulted in a chimeric chemokine that, when compared to CCL15(Δ26), exhibited biased agonism towards cAMP inhibition relative to βArr recruitment. Similarly, truncating the N-terminus of CCL15(Δ26) by two residues resulted in biased agonism in comparison to CCL15(Δ26). These results confirm that the N-terminal regions of chemokines play a role in preferential activation of specific signaling pathways via CCR1. By extension, we anticipate that the N-terminal regions of cognate chemokines are likely to also influence biased agonism at other chemokine receptors.

Our observation that CCL15(Δ26) and CCL15(Δ28) differentially activate CCR1-coupled signaling pathways is the first observation that variation of chemokine N-terminal length gives rise to biased agonism. Many chemokines undergo N-terminal processing by endogenous proteases [32,33]. N-terminal truncation may increase or decrease the potency and/or efficacy of the chemokines at their cognate receptors. Our results suggest that, at least in some cases, such truncations are likely to also alter the relative activation of different signaling pathways. Alteration of pathway selectivity resulting from N-terminal truncation is a previously unrecognized mechanism by which chemokine-receptor networks may be regulated.

**Figure 5:**
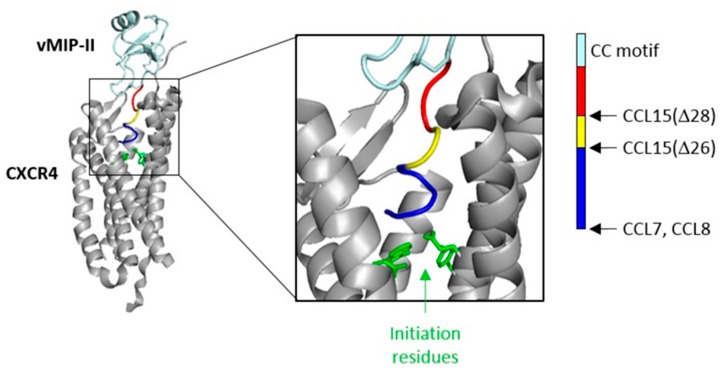
Position of chemokine N-terminal region in the receptor-binding pocket. Full (**left**) and zoom-in (**center**) views of the structure (PDB code: 4RWS) of the complex between CXCR4 (gray ribbons; side chains of signal initiation residues shown as green sticks) and vMIP-II (light cyan ribbons with disulfides as sticks; N-terminal region highlighted in: blue, residues 1–5; yellow, residues 6, 7; and red, residues 8–10). (**Right**) Bar showing the relative lengths of the N-terminal regions for CCR1 cognate chemokines described in this study.

Finally, our observation of biased agonism for CCL15(Δ28) in comparison to CCL15(Δ26) has implications for structural models of chemokine receptor activation. The structure of the viral inhibitory CC chemokine vMIP-II bound to receptor CXCR4 [24] shows that the N-terminal region of the chemokine binds deep into a pocket defined by the interior surfaces of the receptor TM helices (Figure 5), where it is able to form interactions with receptor residues proposed to be required for initiation of receptor activation. The N-terminal region of vMIP-II is the same length (ten residues) as those of CCL7 and CCL8, and just one residue longer than those of CCL3 and CCL5. Thus, these chemokines are likely to penetrate the CCR1 binding pocket to a similar depth as observed in the vMIP-II–CXCR4 complex, forming interactions with these “initiation residues”. On the other hand, the N-terminal regions of CCL15(Δ26) and CCL15(Δ28) are five and seven residues shorter, respectively. If the N-terminal region of CCL15(Δ26) is fully extended, it may be able to extend into the binding pocket almost as far as that of vMIP-II. However, the N-terminal region of CCL15(Δ28) is certainly not long enough to reach the same depth and interact with the same initiation residues. Thus, this shorter form of CCL15 is likely to initiate CCR1 activation by binding to receptor residues closer to the extracellular side of the receptor. We speculate that the differential agonism of CCL15(Δ26) and CCL15(Δ28) may result from them interacting with different groups of receptor residues to initiate signaling. 

## 4. Materials and Methods

### 4.1. Materials

Coelenterazine h was purchased from NanoLight (Pinetop, AZ, USA). CCL15(Δ28) and CCL15(N-CCL7) genes were obtained from GenScript (Piscataway, NJ, USA). Unless otherwise noted, all the other reagents were purchased from Sigma-Aldrich (Castle Hill, Australia). 

### 4.2. Chemokine Expression and Purification

All chemokines and chimeras were expressed and purified as described [26,34]. Briefly, the N-terminal His_6_-tagged fusion protein was expressed as inclusion bodies in *E. coli*, denatured, purified by Ni^2+^-affinity chromatography, refolded, the His_6_-tag removed proteolytically, and the protein further purified by size-exclusion chromatography. Purity was evaluated using SDS-PAGE and protein identity was validated by mass spectrometry. CCL8, for which the Lys-46 allele was used, contained the Pro-8→Ala mutation to ensure that this protein is monomeric.

### 4.3. Mammalian Cell Culture and Binding and Signaling Assays

Flp-In™ T-REx™ 293 cells (Invitrogen, Carlsbad, CA, USA) that stably express N-terminally His_6_-tagged cMyc-tagged human CCR1 were obtained and maintained as described [28] and used for all assays, with the exception of βArr2 recruitment. Cell membranes were prepared and used for competitive radioligand binding assays (radioligand 50 pM ^125^I-CCL3; sample incubation for 2 h at 37 °C) according to published procedures [28]. Signaling assays to assess recruitment of βArr2, G protein activation, inhibition of forskolin-induced cAMP production, and phosphorylation of ERK1/2 were all performed as described previously [28]. Briefly, recruitment of βArr2 was monitored using Flp-In™ T-REx™ 293 cells transiently transfected to express CCR1 fused to RLuc8 and βArr2 fused to YFP [35]; ligands were added 5 min after coelenterazine h and then cells were incubated for 10 min (unless otherwise noted) in the dark at 37 °C before measurement of bioluminescence resonance energy transfer (BRET). G protein activation was measured using cells transiently transfected with DNA encoding G_αi_, G_β-Venus(C-terminus)_, G_γ-Venus(N-terminus)_, and masGRK3-ct-Rluc [36] in the ratio 2:1:1:1; cells were incubated with coelenterazine h for 5 min then the indicated concentrations of ligands for 10 min (unless otherwise noted) prior to BRET measurement. Inhibition of forskolin-induced cAMP production was evaluated using cells transfected with the cAMP BRET biosensor using YFP-Epac-RLuc (CAMYEL) [37] and incubation times of 5 min with coelenterazine h, followed by 5 min with chemokine at the indicated concentration, followed by 5 min (unless otherwise noted) with forskolin (10 µM). ERK1/2 phosphorylation was evaluated using the PerkinElmer AlphaScreen^®^ SureFire^®^ phospho-ERK 1/2 (Thr-202/Tyr-204) (Waltham, MA, USA) and an incubation time of 5 min (unless otherwise noted) with chemokine at the indicated concentration, before removal of the medium and cell lysis. 

### 4.4. Data Analysis and Statistics

All experiments were performed at least three times independently. Data points presented are the mean and error bars are the standard error of the mean (SEM) from the independent measurements. Data were analyzed using Prism 6.0 (GraphPad Software Inc., San Diego, CA, USA) and fitted to established equations for competitive binding or concentration-response signaling. Briefly, competitive radioligand binding data were fitted to the equation:

(1) $$Y=bottom+\;\frac{top-bottom}{1+\;10^{\left(X-\log IC_{50}\right)}}$$

where *X* is the concentration of competitor (chemokine); *Y* is the percentage specific binding; top and bottom represent the maximum and minimum asymptotes, respectively; and *IC_50_* is the concentration of competitor that inhibited half of the radioligand binding.

Concentration-response signaling data, after appropriate normalization, were fitted to the equation:

(2) $$Y=bottom+\;\frac{top-bottom}{1+\;10^{\left(\log EC_{50}-\log\left[A\right]\right)}}$$

where [A] is the molar concentration of agonist; top and bottom represent the maximum and minimum asymptotes, respectively; and *EC_50_* is the molar concentration of agonist that gives a response half way between the maximum and minimum asymptotes.

For evaluation of biased agonism, concentration-response data for all chemokines for each pathway were fitted globally to the operational model of agonism of Black and Leff [30], and bias parameters determined as described previously [26,31]. This analysis yielded the transduction coefficient for each chemokine, log*(τ/K_A_)*, where τ is an index of the signaling efficacy of the agonist for the relevant pathway and *K_A_* is the equilibrium dissociation constant of the agonist for the form of the receptor coupled to the relevant signaling pathway. The transduction coefficient of CCL15(Δ26), the reference agonist, was subtracted from those of each other chemokine to yield Δlog(*τ*/*K_A_*) values, thereby eliminating cell-dependent and assay-dependent effects. The relative bias between two signaling pathways was then calculated for each chemokine by subtracting the Δlog(*τ*/*K_A_*) of one pathway from that of the other, giving ΔΔlog(*τ*/*K_A_*) or LogBias values. LogBias values of zero indicate that there is no biased agonism between the pathways.

For statistical analyses, *EC_50_* and *IC_50_* were estimated as their logarithms (*pEC_50_* and *pIC_50_*, respectively) to enable valid statistical comparison [38], using multiple *t* test with Holm–Sidak correction or one- and two-way ANOVA, as stated in figure legends. Significance is indicated as * for *p* < 0.05, ** for *p* < 0.01 and *** for *p* < 0.001 for the comparison graphs.

## Figures and Tables

**Table 1:** Affinity, potency, and efficacy of three wild type chemokines at CCR1 ^1^.

Assay	CCL15(Δ26)	CCL8	CCL7
Radioligand binding (*pIC_50_*)	10.1 ± 0.1 (0.093)	8.6 ± 0.1 (2.3) ***	9.5 ± 0.1(0.33) **
βArr2 recruitment (*pEC_50_*)	8.6 ± 0.1 (2.6)	7.0 ± 0.1 (110) ***	8.3 ± 0.1 (5.2)
βArr2 recruitment (*E_max_*, %)	94.3 ± 4	98.4 ± 7	43.1 ± 2 ***
G protein activation (*pEC_50_*)	9.0 ± 0.2 (1.0)	7.5 ± 0.1 (30) ***	7.9 ± 0.1 (14) **
G protein activation (*E_max_*, %)	110.6 ± 7	172.2 ± 9 **	160.3 ± 8 **
cAMP inhibition (*pEC_50_*)	8.6 ± 0.1 (2.8)	7.3 ± 0.1 (48.0) ***	8.0 ± 0.1 (9.5) **
cAMP inhibition (*E_max_*, %)	28.7 ± 2	46.0 ± 3 **	48.8 ± 2 **
ERK1/2 phosphorylation (*pEC_50_*)	8.6 ± 0.2 (2.3)	7.2 ± 0.1 (65.5) ***	8.0 ± 0.1 (9.6) *
ERK1/2 phosphorylation (*E_max_*, %)	25.4 ± 1	20.8 ± 1 *	30.4 ± 1 *

^1^*pEC_50_* and *pIC_50_* values are the negative log of *EC_50_* and *IC_50_* values, respectively, in molar units. *E_max_* values are relative to the positive control or the reference ligand CCL15(Δ26). Data are means ± SEM of at least three independent experiments, performed in duplicate. The corresponding *EC_50_* or *IC_50_* values (in nM) are shown in parentheses. * *p* < 0.05, ** *p* < 0.01, *** *p* < 0.001, relative to CCL15(Δ26). Statistical analysis was performed using one-way ANOVA with Holm–Sidak’s multiple-comparison.

**Table 2:** Rank orders of potency and maximal effect for CCR1 activation by chemokines ^1^.

Assay	Order of Potency (*pEC_50_*)	Order of Maximal Effect (*E_max_*)
βArr recruitment	CCL15 ~ CCL7 > CCL8	CCL15 ~ CCL8 > CCL7
G protein activation	CCL15 > CCL7 ~ CCL8	CCL7 ~ CCL8 > CCL15
cAMP inhibition	CCL15 ~ CCL7 > CCL8	CCL7 ~ CCL8 > CCL15
ERK1/2 phosphorylation	CCL15 ~ CCL7 > CCL8	CCL7 > CCL15 > CCL8

^1^ CCL15 ranks are for CCL15(Δ26).

**Table 3:** Affinity, potency, and efficacy of chemokine N-terminal variants at CCR1 ^1^.

Assay	CCL15(Δ26)	CCL15(Δ28)	CCL15(N-CCL7)	CCL7
Radioligand binding (*pIC_50_*)	10.6 ± 0.2 (0.028)	10.1 ± 0.2 (0.074)	9.3 ± 0.2 (0.54) **	8.9 ± 0.2 (1.2) ***
βArr2 recruitment (*pEC_50_*)	8.6 ± 0.1 (2.6)	8.9 ± 0.1 (1.2)	8.9 ± 0.1 (1.1)	8.3 ± 0.1 (5.2)
βArr2 recruitment (*E_max_*, %)	94.3 ± 4	73.5 ± 4 **	71.0 ± 3 **	43.1 ± 2 ***
Gα_i1_ activation (*pEC_50_*)	9.0 ± 0.2 (1.0)	8.6 ± 0.3 (2.3)	9.2 ± 0.3 (0.7)	7.9 ± 0.1 (13.6) *
Gα_i1_ activation (*E_max_*, %)	110.6 ± 7	86.2 ± 9	91.2 ± 8	160.3 ± 8 **
Gα_i2_ activation (*pEC_50_*)	8.9 ± 0.3 (1.2)	8.9 ± 0.2 (1.3)	8.8 ± 0.2 (1.5)	7.6 ± 0.1 (26.4) **
Gα_i2_ activation (*E_max_*, %)	114.0 ± 10	140.4 ± 8	124.5 ± 9	195.5 ± 9 ***
cAMP inhibition (*pEC_50_*)	8.6 ± 0.1 (2.7)	9.4 ± 0.2 (0.4) *	9.7 ± 0.2 (0.2) **	8.0 ± 0.1 (9.5) *
cAMP inhibition (*E_max_*, %)	28.7 ± 2	35.7 ± 2	35.5 ± 2	48.8 ± 2 ***
ERK1/2 phosphorylation (*pEC_50_*)	8.6 ± 0.2 (2.3)	8.7 ± 0.1 (2.1)	8.9 ± 0.1 (1.3)	8.0 ± 0.1 (9.6) *
ERK1/2 phosphorylation (*E_max_*, %)	25.4 ± 1	28.3 ± 1	28.2 ± 1	30.4 ± 1 *

^1^*pEC_50_* and *pIC_50_* values are the negative log of *EC_50_* and *IC_50_* values, respectively, in molar units. *E_max_* values are relative to the positive control or the reference ligand CCL15(Δ26). Data are means ± SEM of at least three independent experiments, performed in duplicate. The corresponding *EC_50_* or *IC_50_* values (in nM) are shown in parentheses. * *p* < 0.05, ** *p* < 0.01, *** *p* < 0.001, relative to CCL15(Δ26). Statistical analysis was performed using one-way ANOVA with Holm–Sidak’s multiple-comparison.

## Abbreviations

βArr	recruitment of β-Arrestin 2
ANOVA	analysis of variance
ATP	adenosine triphosphate
BRET	bioluminescence resonance energy transfer
cAMP	3′,5′-cyclic adenosine monophosphate
CCL	C-C motif chemokine ligand
CCR	C-C motif chemokine receptor
ERK1/2	extracellular signal regulated kinases 1 and 2
GPA	G protein activation
GPCR	G protein-coupled receptor
HCC-2	hemofiltrate C-C motif chemokine 2
HEK	human embryonic kidney
MCP	monocyte chemoattractant protein
MIP	macrophage inflammatory protein
p*EC_50_*	−log_10_(*EC_50_*) where *EC_50_* is the concentration required for 50% activation
pERK	phosphorylated ERK
p*IC_50_*	−log_10_(*IC_50_*) where *IC_50_* is the concentration required for 50% inhibition
Rluc	Renilla luciferase
SDS-PAGE	Sodium dodecyl sulfate polyacrylamide gel electrophoresis
TM	transmembrane
T-Rex	tetracycline-regulated expression
YFP	yellow fluorescent protein

## Appendix A

**Table A1:** Transduction coefficients Log(τ/K_A_) for wild type chemokines ^1^.

Assay	CCL15(Δ26)	CCL8	CCL7
βArr2	8.4 ± 0.04	6.8 ± 0.05	7.1 ± 0.2
GPA αi1	8.6 ± 0.04	7.4 ± 0.05	7.9 ± 0.3
cAMP	8.1 ± 0.07	7.3 ± 0.02	7.9 ± 0.2
pERK	8.4 ± 0.2	7.1 ± 0.1	7.6 ± 0.1

^1^ Data are the mean ± SEM from at least three independent experiments performed in duplicate. Each independent experiment was fitted with the operational model of agonism to obtain transduction coefficients that were then averaged.

**Table A2:** Transduction coefficients Log(τ/K_A_) for chemokine N-terminal variants ^1^.

Assay	CCL15(Δ26)	CCL15(Δ28)	CCL15(N-CCL7)	CCL7
βArr2	8.7 ± 0.1	8.8 ± 0.2	8.8 ± 0.1	7.5 ± 0.1
GPA α_i1_	8.7 ± 0.07	8.4 ± 0.2	9.0 ± 0.2	7.8 ± 0.1
GPA α_i2_	8.3 ± 0.1	8.6 ± 0.1	8.5 ± 0.1	7.6 ± 0.07
cAMP	8.4 ± 0.1	8.9 ± 0.1	9.1 ± 0.1	7.9 ± 0.1
pERK	8.7 ± 0.1	8.6 ± 0.1	8.8 ± 0.1	8.0 ± 0.1

^1^ Data are the mean ± SEM from at least three independent experiments performed in duplicate. Each independent experiment was fitted with the operational model of agonism to obtain transduction coefficients that were then averaged. Parameters listed both here and in Table A1 were determined for these tables in two different sets of experiments and are not significantly different between the two.
